# Is Minimal Change Disease Associated with Prostate Cancer or Is Age Just a Number?

**DOI:** 10.3390/reports7030070

**Published:** 2024-08-13

**Authors:** Patrícia Kleinová, Matej Vnučák, Karol Graňák, Monika Beliančinová, Tímea Blichová, Ivana Dedinská

**Affiliations:** 1 Transplant-Nephrology Department, University Hospital Martin, Kollárova 2, 03601 Martin, Slovakia; kleinova.pata@gmail.com (P.K.); granak.k@gmail.com (K.G.); beliancinova.monika@gmail.com (M.B.); timea.blichova@gmail.com (T.B.); ivana.dedinska@uniba.sk (I.D.); 2 Department of Internal Medicine I, Jessenius Medical Faculty, Comenius University, 03601 Martin, Slovakia

**Keywords:** prostate cancer, minimal change disease, nephrotic syndrome

## Abstract

Background: Prostate cancer is the most common malignancy in men. Secondary nephrotic syndrome, a feature of paraneoplastic syndrome, occurs in 11% of cases and is mainly caused by membranous glomerulopathy. The association between minimal change disease and prostate cancer is rare. Only one cause has been described in the available literature. Case presentation: We present the case of a 77-year-old patient who was admitted to our department with stage 3 acute kidney injury and with nephrotic syndrome with anasarca (creatinine: 168 µmol/L, eGFR: 33 mL/min/1.73 m^2^, albumin: 18.5 g/L, total cholesterol: 6.86 mmol/L, urine albumin creatinine ratio: 812.7 mg/mmol). In the differential diagnosis of nephrotic syndrome, looking for a secondary cause is essential, so the parainfectious causes of nephrotic syndrome were excluded. An elevated prostate-specific antigen (10.69 ng/L) was found when screening for oncological causes, and prostate adenocarcinoma was identified on biopsy. A renal biopsy was then performed with a finding of minimal change disease. Despite the generally accepted guidelines of prostate carcinoma in that stage and age of the patient being watchful waiting, antiandrogen therapy was started with the cooperation of a urologist. There was a significant improvement in renal parameters in the patient (creatinine: 87 µmol/L, eGFR: 73 mL/min/1.73 m^2^, albumin: 33.4 g/L, urine albumin creatinine ratio: 27.6 mg/mmol). Conclusion: This case shows the importance of multidisciplinary cooperation in the treatment of secondary causes of nephrotic syndrome. In the case of proven paraneoplastic syndrome, it is necessary to start treating the malignancy; however, in general, a conservative approach without treatment is recommended.

## 1. Introduction

Prostate cancer (PC) is the second most commonly diagnosed malignancy in men worldwide, after skin cancer [1]. Secondary nephrotic syndrome (NS) is a rare clinical presentation of prostate cancer and is a part of paraneoplastic syndrome. Renal involvement is a result of tumour cell products such as tumour antigens, hormones, and cytokines [2,3]. The most frequent paraneoplastic glomerulopathy is secondary membranous nephropathy [4].

Minimal change disease (MCD) is a podocytopathy, predominantly (80–90%) causing primary nephrotic syndrome in children under 10 years of age.

In adults, it accounts for 10–25% of adult NS and can be associated with acute kidney injury (AKI). The sudden development of NS is typical for MCD. The response to treatment is excellent, but the probability of relapse is high. The recommended first-line treatment is high-dose oral glucocorticoids (1 mg/kg, maximum 80 mg per day) for a maximum duration of 16 weeks. Cyclophosphamide, calcineurin inhibitors, or mycophenolic acid analogues are recommended for the treatment of relapsing MCD or for patients in whom oral corticosteroids are contraindicated [5].

Here, we present the rare case of a patient with prostate cancer with nephrotic syndrome associated with minimal change disease, where only one case has been described in the available literature [6].

## 2. Detailed Case Description

A 77-year-old male was hospitalised in the Transplant-Nephrology department at University Hospital Martin with AKI stage III (creatinine: 168 µmol/L and anuria) according to Kidney Disease Improving Global Outcomes (KDIGO) and with nephrotic syndrome with anasarca.

Dyspnoea, a murmur in the precordium, and generalised pitting oedema were present on physical examination.

**Figure 1:**
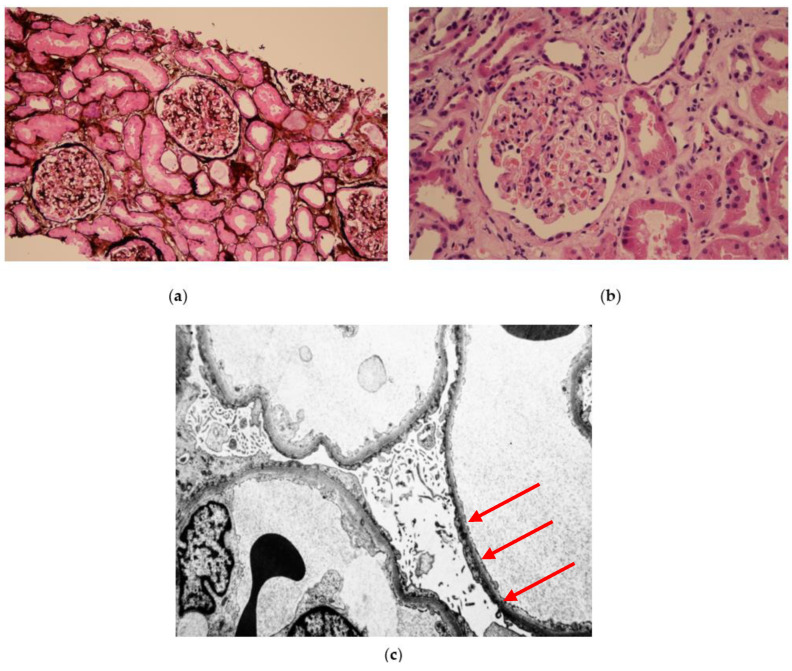
Histological examination of renal biopsy. (**a**) Light microscopy with periodic acid-methenamine silver staining (magnification ×100): no changes in the glomerular basal membrane; (**b**) light microscopy with haematoxylin eosin staining (magnification ×400): no changes are observed in the glomeruli; (**c**) electron microscopy (magnification ×5000): extensive foot processes effacement (red arrows), no electron dense deposits or segmental sclerosis, and normal glomerular basement membrane thickness.

Laboratory examinations were performed on admission, and nephrotic syndrome was diagnosed [creatinine: 168 µmol/L, estimated glomerular filtration rate (eGFR): 33 mL/min/1.73 m^2^, albumin: 18.5 g/L, total cholesterol: 6.86 mmol/L, urine albumin/creatinine ratio: 812.7 mg/mmol] (shown in Table 1). eGFR was calculated according to the Chronic Kidney Disease Epidemiology Collaboration (CKD-EPI) formula. Within the differential diagnosis of primary nephrotic syndrome, a renal biopsy was performed. The histopathology showed ten glomeruli, and on light microscopy, any pathomorphology abnormalities of the glomeruli were not detected. Immunofluorescence studies did not reveal any deposits, and complete fusion of podocyte pedicels and villous transformation of podocyte cytoplasm was found on electron studies (shown in Figure 1). Based on the microscopic findings, the renal biopsy result was concluded as MCD. High-dose oral glucocorticoids (40 mg per day) with gradual weaning were administered due to biopsy-proven MCD. Supportive therapy for nephrotic treatment (albumin, loop intravenous diuretics, hypolipidemics, and antiplatelet therapy) was administered. Forced diuresis did not have an effect. During the patient’s hospitalisation, AKI progressed with the retention of nitrogenous substances, for which haemodialysis was initiated.

**Figure 2:**
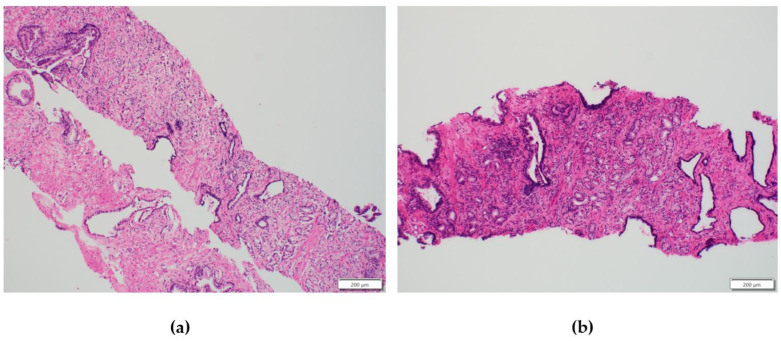
Histological examination of prostate biopsy. (**a**) H&E staining (magnification ×100): adenocarcinoma formed by a mixture of glands with a preserved lumen (Gleason growth pattern 3) and glands with an indistinct, minimally marked lumen (Gleason growth pattern 4). (**b**) H&E staining (magnification ×100): adenocarcinoma formed by the predominance of Gleason growth pattern 3 with minimal involvement of growth pattern 4: Gleason score 7 (3 + 4).

In the differential diagnostic algorithm, an infectious cause was excluded with negative serology (hepatitis A virus, hepatitis B virus, hepatitis C virus, toxoplasma, herpes simplex, varicella zoster virus, cytomegalovirus, syphilis, HIV) for the secondary causes of the nephrotic syndrome. Complement levels were within the physiological range; autoimmune antibodies were not detected including anti-phospholipase A2 receptor antibodies (antiPLA2r), and the kappa/lambda ratio was elevated but within the renal reference range (0.37–3.1). A paraneoplastic cause was demonstrated by an elevated prostate-specific antigen (PSA) level, followed by the detection of acinar adenocarcinoma of the prostate (pT1cN0M0) with Gleason score 7 (3 + 4) on biopsy (shown in Figure 2). X-ray oncology screening was performed without the detection of metastasis. According to the American Joint Committee on Cancer (AJCC), the patient was stage IIB (intermediate risk group). Considering the results of both histological examinations, we assumed the occurrence of secondary MCD with underlying oncological diseases. A urologist was consulted with the findings, where a treatment strategy of “watchful waiting” was recommended for the given stage and due to the patient’s age [3].

**Figure 3:**
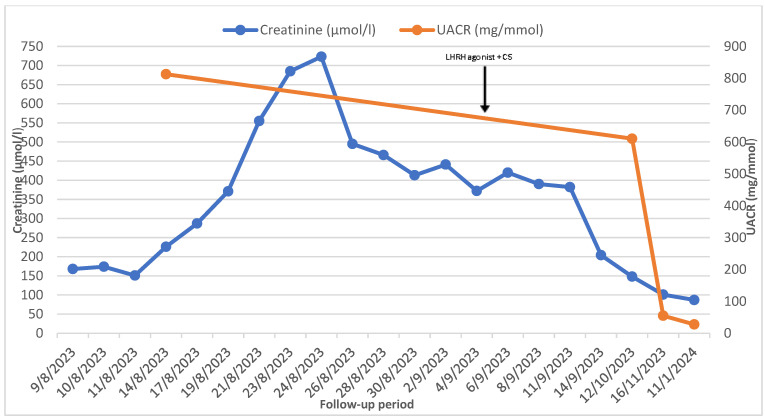
Clinical course before and after LHRH agonist and oral glucocorticoids administration; UACR—urine albumin/creatine ratio, LHRH—luteinising hormone-releasing hormone, CSs—corticosteroids.

**Figure 4:**
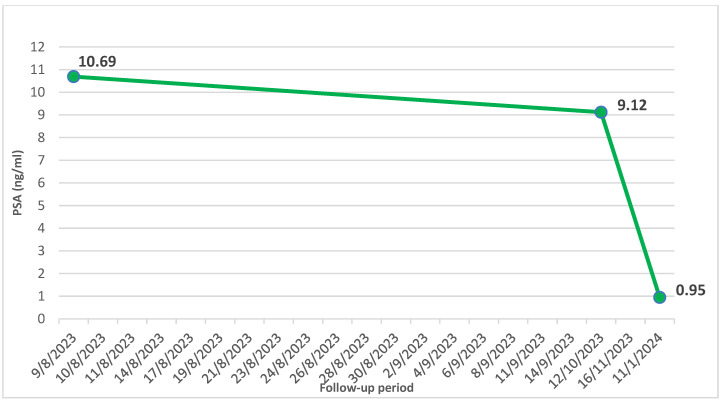
The development of a prostate-specific antigen after LHRH agonist administrations; PSA—prostate-specific antigens, LHRH—luteinising hormone-releasing hormone.

Based on our repeated request, despite the recommendations, antiandrogen therapy with a subcutaneous luteinising hormone-releasing hormone (LHRH) agonist was commenced by the urologist. After two doses of the LHRH agonist, high-dose oral glucocorticoids were administered, and the patient experienced a significant improvement in his renal parameters (creatinine: 87 μmol/L, eGFR: 73 mL/min/1.73 m^2^, urinary albumin/creatinine ratio: 27.6 mg/mmol), PSA (0.95 ng/L), and a restoration of diuresis without the need for haemodialysis treatment (as shown in Figure 3 and Figure 4).

## 3. Discussion

The primary causes of nephrotic syndrome are membranous nephropathy, minimal change disease, and focal glomerulosclerosis. MCD is commonly found in children (70–90%). On the other hand, membranous nephropathy (MN) is a typical cause of idiopathic nephrotic syndrome in adults [2,4].

Secondary NS can be caused by autoimmune disease (such as lupus erythematosus or ANCA-associated vasculitis), diabetes mellitus, infection, and malignancy [4]. NS, as a feature of paraneoplastic syndrome, is found in 11% of patients with malignancy [2]. The most common paraneoplastic glomerulopathy is membranous glomerulopathy associated with a solid tumour, mostly carcinomas of the lung or of the gastrointestinal tract. On the other hand, minimal change disease is associated with Hodgkin’s lymphoma [5]. Here, we present a case of MCD associated with prostate cancer.

The clinical presentation of MCD is nephrotic syndrome with proteinuria greater than 3 to 3.5 g/24 h. A renal biopsy is necessary to diagnose MCD. Electron microscopy shows the fusion of the pedicels of podocytes but no electron-dense deposits. Light microscopy demonstrates no glomerular lesion or minimal mesangial prominence, and immunofluorescence is usually negative. However, immunoglobulin (Ig) M, C3, or C1q can be positive [2,5]. Our patient met all the conditions of nephrotic syndrome with biopsy-verified MCD, without the detection of deposits in immunofluorescence.

Prostate cancer is the most common malignancy (except for skin cancer) in men, and it has been associated with MN [3,7]. The treatment of PC strongly depends on the stage of the cancer. The most widely used staging system for prostate cancer is the AJCC TNM system (tumour, node, metastasis), which is based on PSA level, grade group (Gleason score), and extent of the cancer (T, N, and M categories). There are four stages of PC (I., II.A-II.C, III.A-III.C, and IV.A-IV.B), and our patient was in the IIB stage [PSA: 10.69 ng/L, Gleason score 7 (3 + 4) and pT1cN0M0], placing him in the intermediate-risk group. In this group, five treatment options are available: radiotherapy, hormone therapy, radical prostatectomy with pelvic lymph node dissection, active surveillance, and observation [3]. Following a consultation with a urologist, he was commenced on hormonal therapy with LHRH agonists due to a strong suspicion of paraneoplastic glomerulopathy caused by PC. LHRH agonists target the pituitary LRHR receptors (LHRH-R), resulting in an increased level of testosterone caused by an initial surge in the luteinising hormone (LH) and follicle-stimulating hormone (FSH). This effect is called tumour flare, but continued overstimulation of LHRH-R decreases the level of testosterone to castration levels [8].

To our knowledge, only Nakano et al. have reported a case where MCD presented as a complication of prostate adenocarcinoma. The case they described followed a similar clinical course, but their management was different. They decided to use combined therapy: hormonal and radiation therapy without high-dose steroid therapy. Moreover, they also prescribed diuretics to improve peripheral oedema. Their patient achieved complete remission of MCD in 2 months [6]. According to KDIGO, complete remission is defined as a reduction in proteinuria to a protein/creatinine ratio (PCR) of <300 mg/g, stable creatinine, and albumin > 35 g/L [5]. 

In contrast, our patient did not respond to diuretics; he required renal replacement therapy. We decided to use a less invasive treatment (LHRH agonists) of PC, but the result was similar, and our patient also achieved complete remission of MCD two months after the first dose of hormonal treatment. His PSA level declined, and there was also a significant decline in nitrogenous end products and in the urine albumin/creatinine ratio (UACR). Furthermore, the patient did not require haemodialysis after the first dose of the LHRH agonist. The result of the treatment strongly supports the link between prostate cancer and MCD.

The pathogenesis of PC is multifactorial. Cytokines of the tumour microenvironment produced by prostate tumour cells, stromal cells, and immune cells play leading roles [6]. Pro-tumour cytokines such as vascular-endothelial growth factor α (VEGF-α) and the interleukins (IL) IL-4, IL-5, IL-6, IL-10, and IL-13 produced by T-helper (Th2) lymphocytes have also been linked to the pathogenesis of MCD [6,9]. Cytokines produced by the TME (tumour microenvironment) reach the glomerular basement membrane (GBM) through circulation, affecting podocytes. This may explain the correlation between the renal manifestation of prostate cancer.

Proangiogenic cytokines such as VEGF-α and IL-8 are essential for angiogenesis and tumour cell metastasis. IL-8 also induces the infiltration of T-cells into the TME. The stimulation of prostate epithelial cells to express 3β-hydroxysteroid dehydrogenase (3b-HSD), which is necessary for generating the active form of androgens, is due to IL-4 and IL-13. IL-10 inhibits anti-tumour responses in PC [6].

One of the latest hypotheses from 2017 claims that the basis of the development of MCD is an imbalance in the subpopulation of suppressor T-lymphocytes with a prevalence of CD8+ lymphocytes and a production of cytokines (IL4, 5, 9, 10, 13) via Th2 lymphocytes [9]. IL-8 increases the metabolism of glycosaminoglycans in the GBM, causes anionic changes in the glomerular basal membrane, and can cause proteinuria [10]. The overexpression of IL-13 is associated with the effacement of podocytes [10]. A 2007 study on rats also supports this association [11]. In addition to those mentioned, substances such as c-mip, hemopexin, and angiopoietin-like 4 can disorganise the cytoskeleton of podocytes, leading to the fusion of podocyte pedicles. Angiopoietin-like 4 is also capable of inducing dyslipidaemia, which is another crucial feature of MCD [10].

## 4. Conclusions

We presented a rare case of a patient with PC-associated MCD. The patient was successfully treated with combined therapy with a LHRH agonist and oral glucocorticoids. Further studies are required to elucidate and confirm the pathogenetic association between PC and MCD. This case shows the importance of multidisciplinary cooperation and the necessity of treating not only MCD itself but also the underlying cause, despite generally valid recommendations in the context of a patient´s quality of life.

## Figures and Tables

**Table 1:** Admission laboratory parameters: TAGs—triacylglycerols, LDL-cholesterol—low-density lipoprotein cholesterol, HDL-cholesterol—high-density lipoprotein cholesterol, Ig—immunoglobulins, WBCs—white blood cells, UACR—urine albumin/creatine ratio, PSA—prostate-specific antigen, eGFR—estimated glomerular filtration rate.

	Baseline	Third Month of Follow-Up	Fifth Month of Follow-Up
**Albumin (g/L)**	18.5 [35.0–52.0]	29.3	33.4
**Creatinine (µmol/L)**	168 [59–104]	101	87
**Urea (mmol/L)**	16.5 [2.8–7.2]	6.6	7.1
**Uric acid (µmol/L)**	518 [208–428]	218	262
**Total protein (g/L)**	41.1 [66.0–83.0]	54.3	61.1
**Total cholesterol (mmol/L)**	6.86 [0.00–5.17]	4.75	4.6
**TAGs (mmol/L)**	2.08 [0.40–1.70]	1.6	1.99
**LDL-cholesterol (mmol/L)**	5.30 [1.00–3.30]	2.62	2.66
**HDL-cholesterol (mmol/L)**	1.20 [1.03–2.00]	1.45	1.04
**Kappa free light chain (mg/L)**	70.34 [3.30–19.40]	-	-
**Lambda free light chain (mg/L)**	29.55 [5.71–26.30]	-	-
**Kappa/Lambda ratio**	2.38 [0.26–1.65]	-	-
**IgG (g/L)**	4.180 [5.400–18.220]	-	-
**IgA (g/L)**	1.954 [1.010–6.450]	-	-
**IgM (g/L)**	2.170 [0.220–2.400]	-	-
**WBCs (10^9^/L)**	7.30 [3.90–10.00]	19.8	14.2
**Thrombocytes (10^9^/L)**	317 [140–400]	282	170
**Haemoglobin (g/L)**	136 [140–179]	113	101
**UACR (mg/mmol)**	812.7 [3–30]	54.9	27.6
**eGFR (ml/min/1.73 m^2^)**	33 [64–104]	61	73
**PSA (ng/L)**	10.69 [0.00–4.00]	9.12	0.95

## Data Availability

The original contributions presented in this study are included in this article; further inquiries can be directed to the corresponding author.
